# 207 ’You can get that on a treadmill, but it’s just not the same kind of thing’. Experiences of VI parkrunners in local green spaces

**DOI:** 10.1093/eurpub/ckae114.141

**Published:** 2024-09-26

**Authors:** Joan Ryan

**Affiliations:** Technological University of Dublin, Dublin, Ireland

## Abstract

**Purpose:**

Access to green spaces is acknowledged to benefit health and wellbeing, but people who are vision impaired (VI) have poorer access both to green spaces and to physical activity. This presentation will explore the experiences of VI participants at parkrun, a mainstream community led 5km event which takes place outdoors.

**Methods:**

This research is part of a wider qualitative study on the experiences of VI participants at parkrun in Ireland. Nine semi structured interviews were carried out on adults with VI who participated at parkrun. Thematic analysis on the data was carried out using the processes of Braun and Clarke.

**Results:**

Participants attributed experiences of being outside in nature to feelings of improved wellbeing, both in terms of their biological health but also improved psychosocial wellbeing and rehabilitation. Being outside in nature, with facilitation and social interaction provided by volunteer sighted guides and fellow parkrunners, was found to enhance the experience of physical activity and the individual agency of the participants.

**Discussion:**

People who are VI face barriers to physical activity in general but also in accessing nature. In addition to the restrictions placed on them by their VI, ableist cultural assumptions of who is welcomed into nature act as further barriers. Participation in the events enhanced the individual agency of the VI participants whereby they could easily access physical activity opportunities in nature and they were incentivised to continue to do so.

**Conclusion:**

This research demonstrates the value VI participants at parkrun placed on their access to physical activity in local green spaces. It demonstrates how a volunteer led community initiative enhanced health and wellbeing for VI participants by prioritising their inclusion in their mainstream outdoor events.

**Support:**

This research is part of a PhD studentship at TU Dublin. Financial Assistance in the form of fees was provided by Topcon and Vision Sports Ireland.

